# Supplementary material for: Characterization of UVA-Induced Alterations to Transfer RNA Sequences
Source: Biomolecules. 2020 Nov 8;10(11):1527. doi: 10.3390/biom10111527 (PMC7695249; doi:10.3390/biom10111527)
Supplement: Supplementary file 1 [file biomolecules-10-01527-s001.pdf]

Supplementary Materials

Characterization of UVA-induced alterations to transfer RNA sequences

Congliang Sun, Patrick A Limbach and Balasubrahmanyam Addepalli*

Rieveschl Laboratories for Mass Spectrometry, Department of Chemistry, University of Cincinnati, Cincinnati, Ohio 45221-0172, United States

* Correspondence: balasual@ucmail.uc.edu

Abstract: Ultraviolet radiation (UVR) adversely affects the integrity of DNA, RNA, and their nucleoside modifications. By employing liquid chromatography tandem mass spectrometry (LC-MS/MS)-based RNA modification mapping approaches, we have identified the transfer RNA (tRNA) regions most vulnerable to photooxidation. Photooxidative damage was consistently observed to the anticodon and variable loop regions in both modified and unmodified sequences of tRNA upon UVA (λ 370 nm) exposure. The extent of oxidative damage measured in terms of oxidized guanosine, however, was higher in unmodified RNA compared to its modified version suggesting an auxiliary role for nucleoside modifications. The type of oxidation product formed in the anticodon stem-loop region varied with the modification type, status, and whether inside or outside the cell during exposure. Oligonucleotide-based characterization of tRNA following UVA-exposure also revealed the presence of novel photoproducts and stable intermediates not observed by nucleoside analysis alone. This approach provides sequence-specific information revealing potential hotspots for UVA-induced damage in tRNAs.

Keywords: UVR, photooxidation, tRNA, posttranscriptional nucleoside modifications, cusativin, RNA modification mapping, RNA oxidation.

Table of contents

Supplemental Figure S1: Representative absorption spectra of unmodified (A), and modified RNA (B) before or after UVA exposure.

Supplemental Figure S2: Detection of U[Gh]UAAAUC>p (*m/z* 1280.8, position 33-40) in the UVA exposed unmodified *in vitro* transcript of tRNA^{Tyr} by LC-MS/MS.

Supplemental Figure S3: Detection of UGCC[Gh]UCAUC>p (*m/z* 1054.2, position 41-50). in the UVA exposed *in vitro* transcript of tRNA^{Tyr} by LC-MS/MS.

Supplemental Figure S4: Detection of AUC[Gh]AC>p (*m/z* 962.2, position 48-53). in the UVA exposed *in vitro* transcript of tRNA^{Tyr} by LC-MS/MS.

Supplemental Figure S5: Detection of UUCGAAG[Gh]UUC>p *m/z* 1177.3 (z=-3, position 54-64) in the UVA exposed *in vitro* transcript of tRNA^{Tyr} by LC-MS/MS.

Supplemental Figure S6: Photooxidative degradative loss of **s^{4}U** in the UVA exposed tRNA^{Tyr} of *E. coli.*

Supplemental Figure S7: Dethiolation of s4U containing oligonucleotide, A[s^{4}U]AG following UVA exposure.

Supplemental Figure S8: Detection of guanosine oxidation product Fapy-G in the tRNA^{Tyr} following UVA exposure.

Supplemental Figure S9: Detection of UGCC[Gh]UCAUCGAC>p**,** *m/z* 1380.8 (z=-3, position 41-52) in the UVA exposed tRNA^{Tyr} of *E. coli* by LC-MS/MS.

Supplemental Figure S10: Detection of U[Q-99]*ψA[ms^{2}A]*AψC>p *m/z* 1313.6 (z=-2, position 33-40) in the UVA exposed tRNA^{Tyr} of *E. coli* by LC-MS/MS.

Supplemental Figure S11: Detection of photooxidative degradation products of queuosine and ms^{2}i^{6}A.

Supplemental Figure S12: Dethiolation of [s^{4}U] in tRNA^{Tyr} isolated from UVA-exposed *E. coli* cells.

Supplemental Figure S13: Detection of dethiolated oligomer pGGUGGGGUUCCC>p in tRNA^{Tyr} isolated from UVA-exposed *E. coli* cells.

Supplemental Figure S14: Decreased levels of [s^{4}U]-containing oligonucleotide U[s^{4}U]AACAAAGp from tRNA$^{\mathbf{Cys}}$ in the UVA exposed *E. coli* cells by LC-MS/MS.

Supplemental Figure S15: Decrease in levels of Q-containing oligonucleotide ACU[Q]UU[t^{6}A]AψCCGp from tRNA$^{\mathbf{Asn}}$ in the UVA exposed and unexposed *E. coli* cells.

Supplemental Figure S16: Decreased levels of mnm^{5}s^{2}U containing oligonucleotide of tRNA^{Glu} in the UVA exposed *E. coli* cells by LC-MS/MS.

Supplemental Figure S17: Decreased levels of m^{2}A containing oligonucleotide from tRNA$^{Arg(ICG)}$ in the UVA exposed compared to unexposed *E. coli* cells.

Supplemental Figure S18: Decreased levels of m^{2}A containing oligonucleotide from tRNA$^{Gln(UUG)}$ in the UVA exposed compared to unexposed *E. coli* cells.

Supplemental Figure S19: Decreased levels of m^2A containing oligonucleotide of $tRNA^{Gln(CUG)}$ in the UVA exposed and unexposed *E. coli* cells.

Supplemental Figure S20: Decreased levels of m^2A containing oligonucleotide from $tRNA^{His(GUG)}$ in the UVA exposed and unexposed *E. coli* cells.

Supplemental Figure S21 Levels of [m^5U]ΨCGp are unaffected between the UVA exposed and unexposed *E. coli* cells.

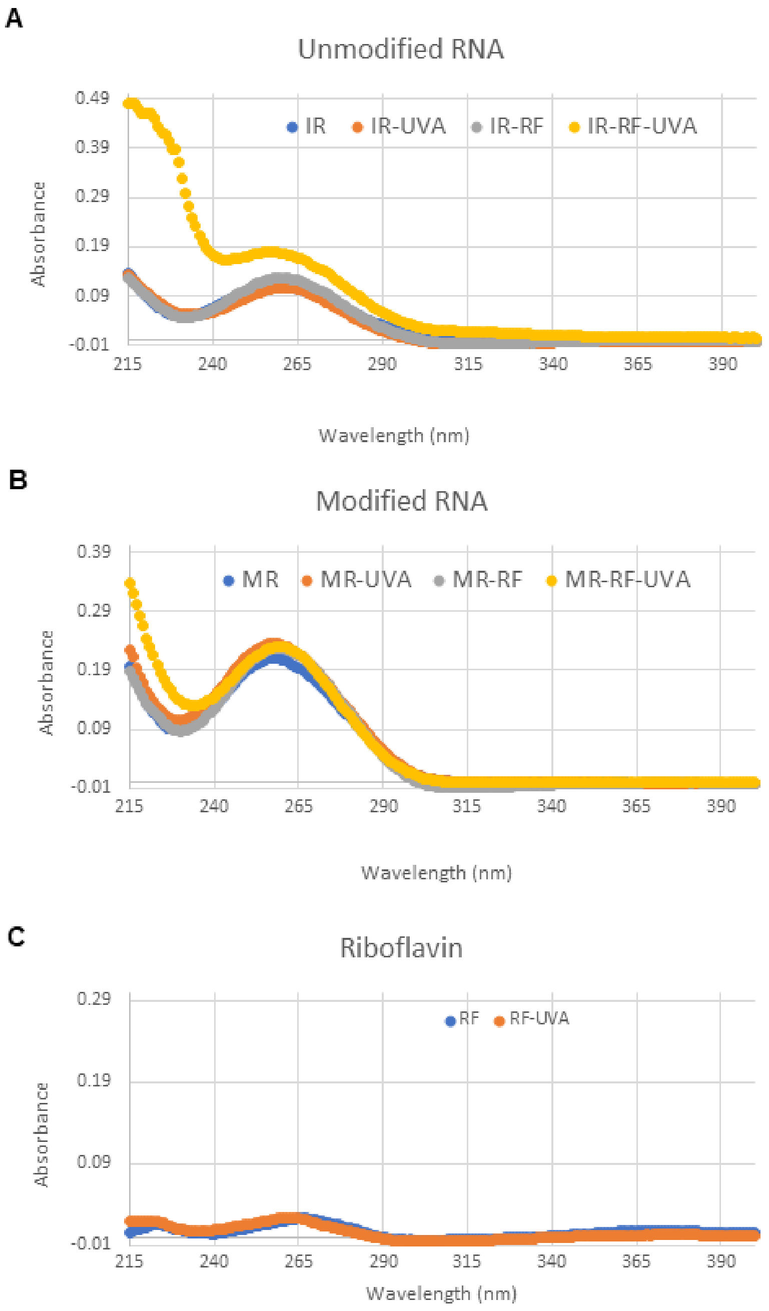

Supplemental Figure S1: Representative absorption spectra of unmodified (A), and modified RNA (B) before or after UVA exposure. The absorbance spectra of riboflavin (RF) for the identical wavelength range is also shown (C). Three µg of transcript RNA (unmodified, labeled as IR) or tRNA (modified, labeled as MR) were scanned for their absorption behavior between 215 and 400 nm wavelength before and after UVA exposure. No significant absorption characteristics were observed at wavelengths above 290 nm for either unmodified (A) or modified RNA (B). UVA treated RNA in the presence of riboflavin (RF) exhibited increased absorption at wavelengths below 240 nm and this increase was prominent for unmodified RNA. Addition of riboflavin did not make a significant contribution to the absorption characteristics. IR: *In vitro* transcript, MR: modified RNA, and RF: riboflavin

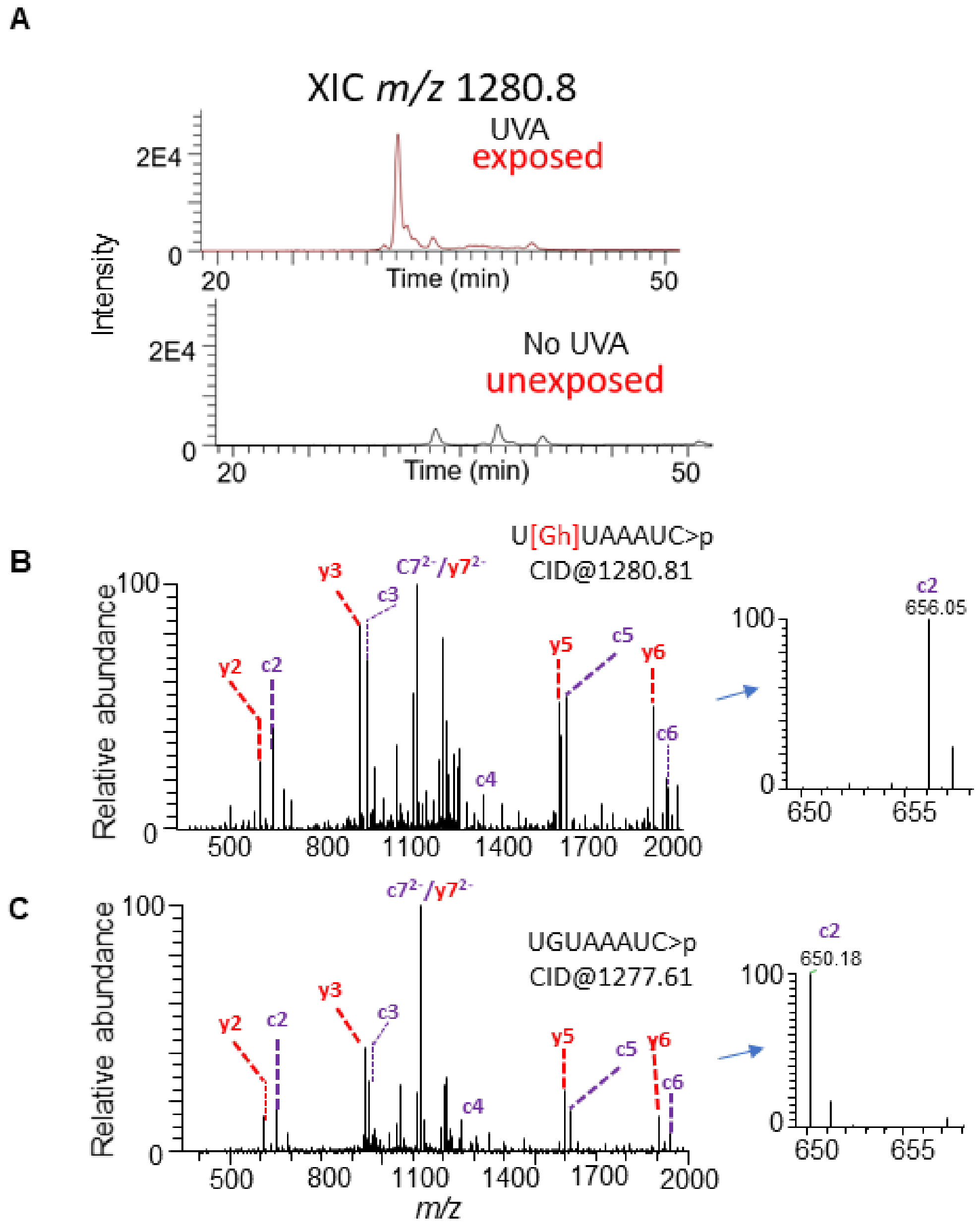

Supplemental Figure S2: Detection of U[Gh]UAAAUC>p (*m/z* 1280.8, position 33-40) in the UVA exposed unmodified *in vitro* transcript of tDNA^{Tyr} by LC-MS/MS. **(A)** Extracted ion chromatogram (XIC) for *m/z* 1280.8 corresponding to U[Gh]UAAAUC>p in the UVA exposed and unexposed samples. (**B**) MS/MS spectrum of *m/z* 1280.81 with sequence informative product ion pattern (cn fragment ion series from 5'-end and yn fragment ion series from 3'-end) of the oligomer, U[Gh]UAAAUC>p (**C**) MS/MS spectrum of *m/z* 1277.6 with sequence informative product ion pattern of the oligomer, UGUAAAUC>p. Note the difference in mass shift of c2 fragment ion in UVA and no UVA exposed samples.

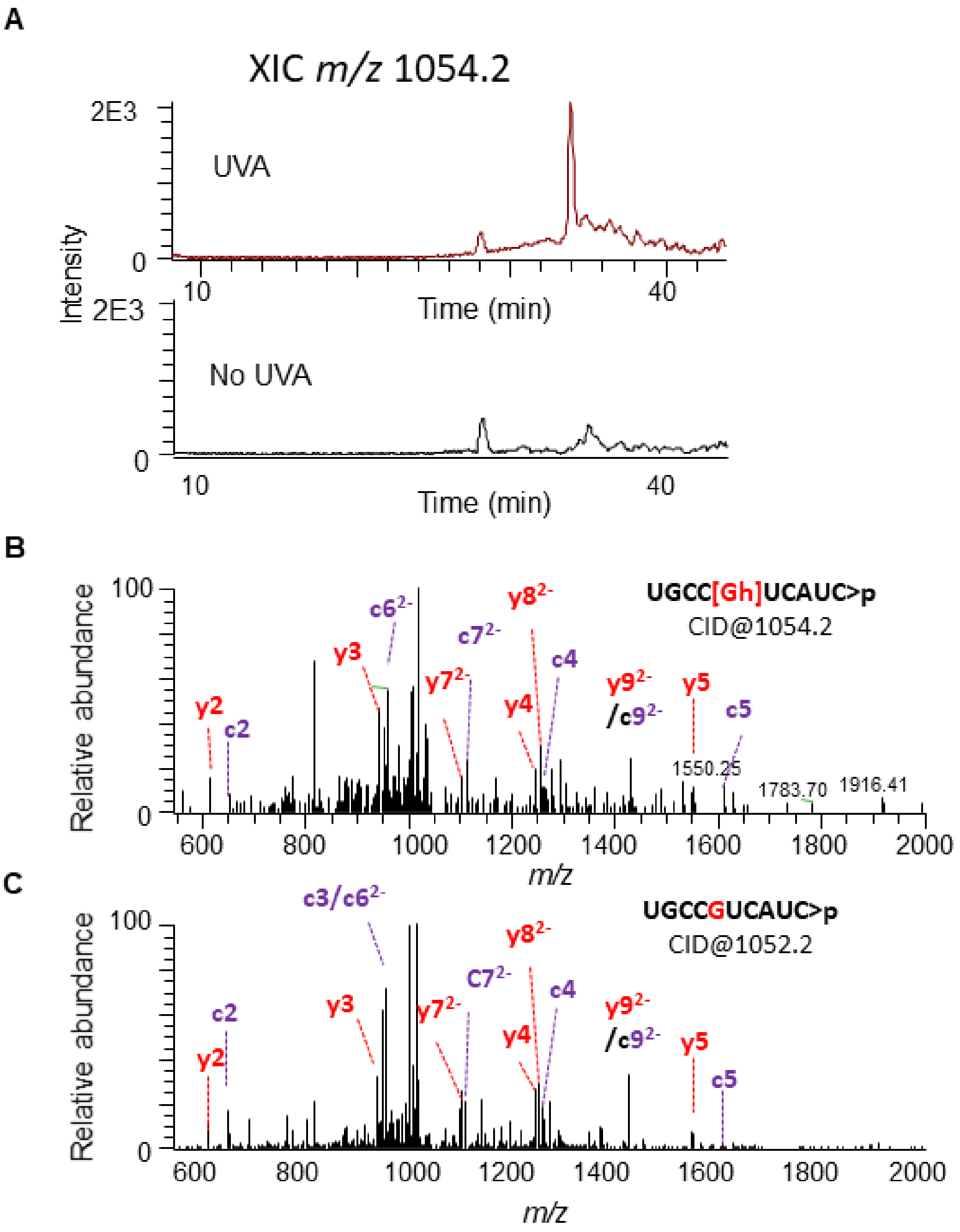

Supplemental Figure S3: Detection of UGCC[Gh]UCAUC>p (*m/z* 1054.2, position 41-50). in the UVA exposed *in vitro* transcript of tDNA^{Tyr} by LC-MS/MS. **(A)** Extracted ion chromatogram (XIC) for *m/z* 1054.2 corresponding to UGCC[Gh]UCAUC>p in the UVA exposed and unexposed samples. (**B**) MS/MS spectrum of *m/z* 1054.2 with sequence informative product ion pattern (cn fragment ion series from 5'-end and yn fragment ion series from 3'-end) of the oligomer, UGCC[Gh]UCAUC>p (**C**) MS/MS spectrum of *m/z* 1052.2 with sequence informative product ion pattern of the oligomer, UGCCGUCAUC>p

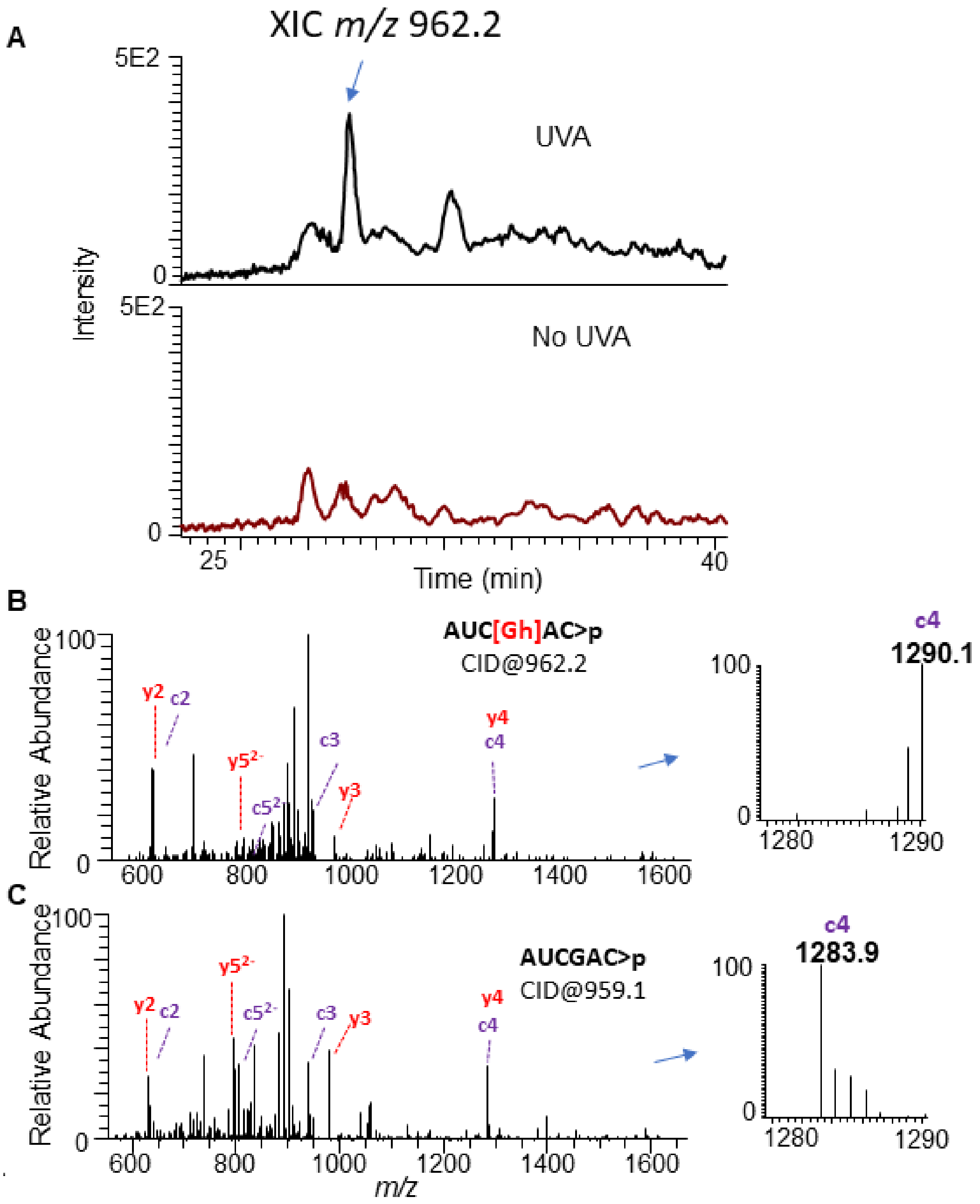

Supplemental Figure S4: Detection of AUC[Gh]AC>p (*m/z* 962.2, position 48-53) in the UVA exposed *in vitro* transcript of tDNA^{Tyr} by LC-MS/MS. **(A)** Extracted ion chromatogram (XIC) for *m/z* 962.2 corresponding to AUC[Gh]AC>p in the UVA exposed and unexposed samples. (**B**) MS/MS spectrum of *m/z* 962.2 with sequence informative product ion pattern (cn fragment ion series from 5'-end and yn fragment ion series from 3'-end) of the oligomer, AUC[Gh]AC>p (**C**) MS/MS spectrum of *m/z* 959.6 with sequence informative product ion pattern of the oligomer, AUCGAC>p**.** Note the difference in mass shift of c4 fragment ion in UVA and no UVA exposed samples.

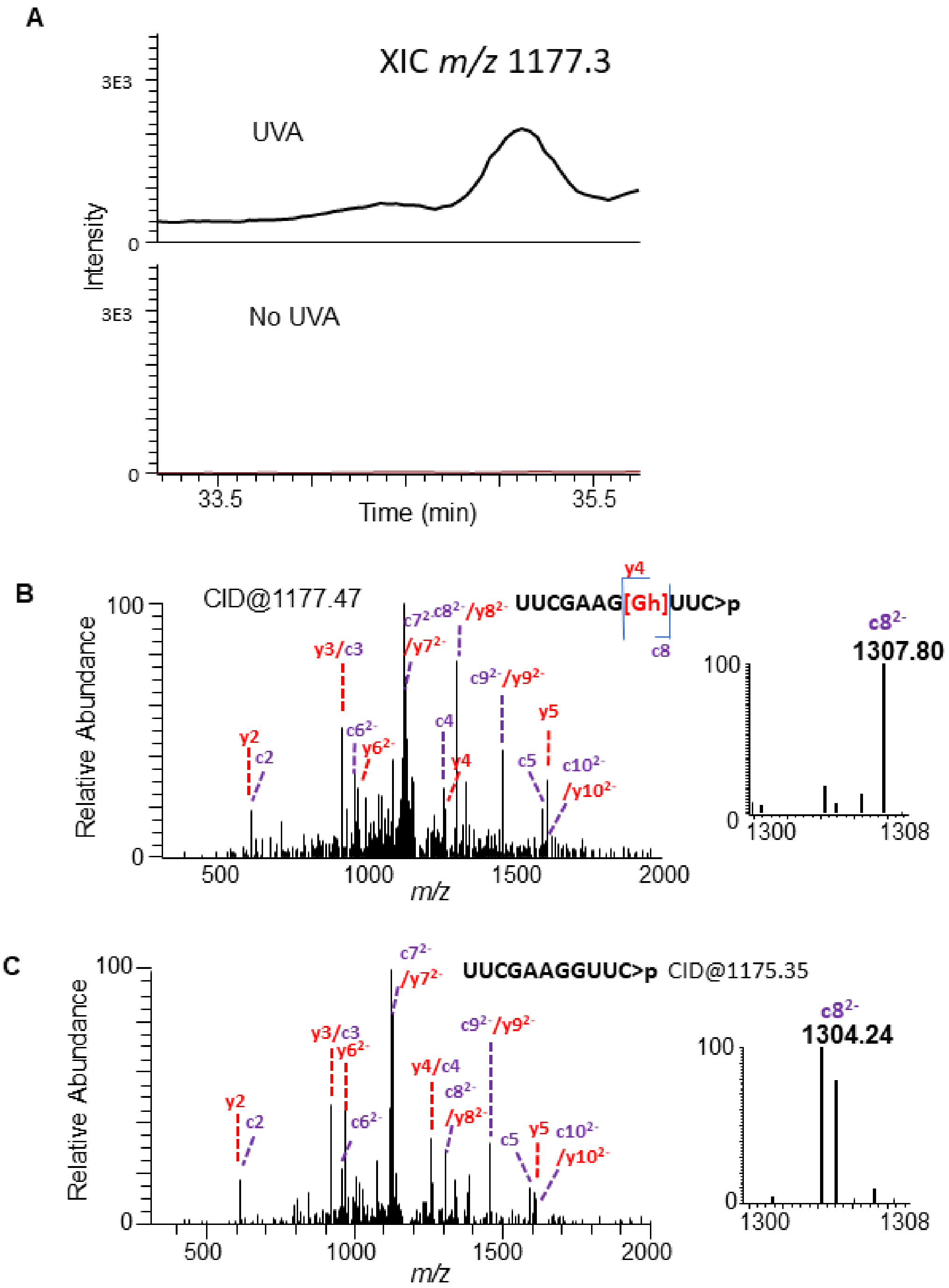

Supplemental Figure S5: Detection of UUCGAAG[Gh]UUC>p *m/z* 1177.3 (z=-3, position 54-64) in the UVA exposed *in vitro* transcript of tDNA^{Tyr} by LC-MS/MS. **(A)** Extracted ion chromatogram (XIC) for *m/z* 1177.3 corresponding to UUCGAAG[Gh]UUC>p in the UVA exposed and unexposed samples. (**B**) MS/MS spectrum of *m/z* 1177.3 with sequence informative product ion pattern (cn fragment ion series from 5'-end and yn fragment ion series from 3'-end) of the oligomer, UUCGAAG[Gh]UUC>p (**C**) MS/MS spectrum of *m/z* 1175.3 with sequence informative product ion pattern of the oligomer, UUCGAAGGUUC>p**.** Note the difference in mass shift of c8 fragment ion in UVA and no UVA exposed samples.

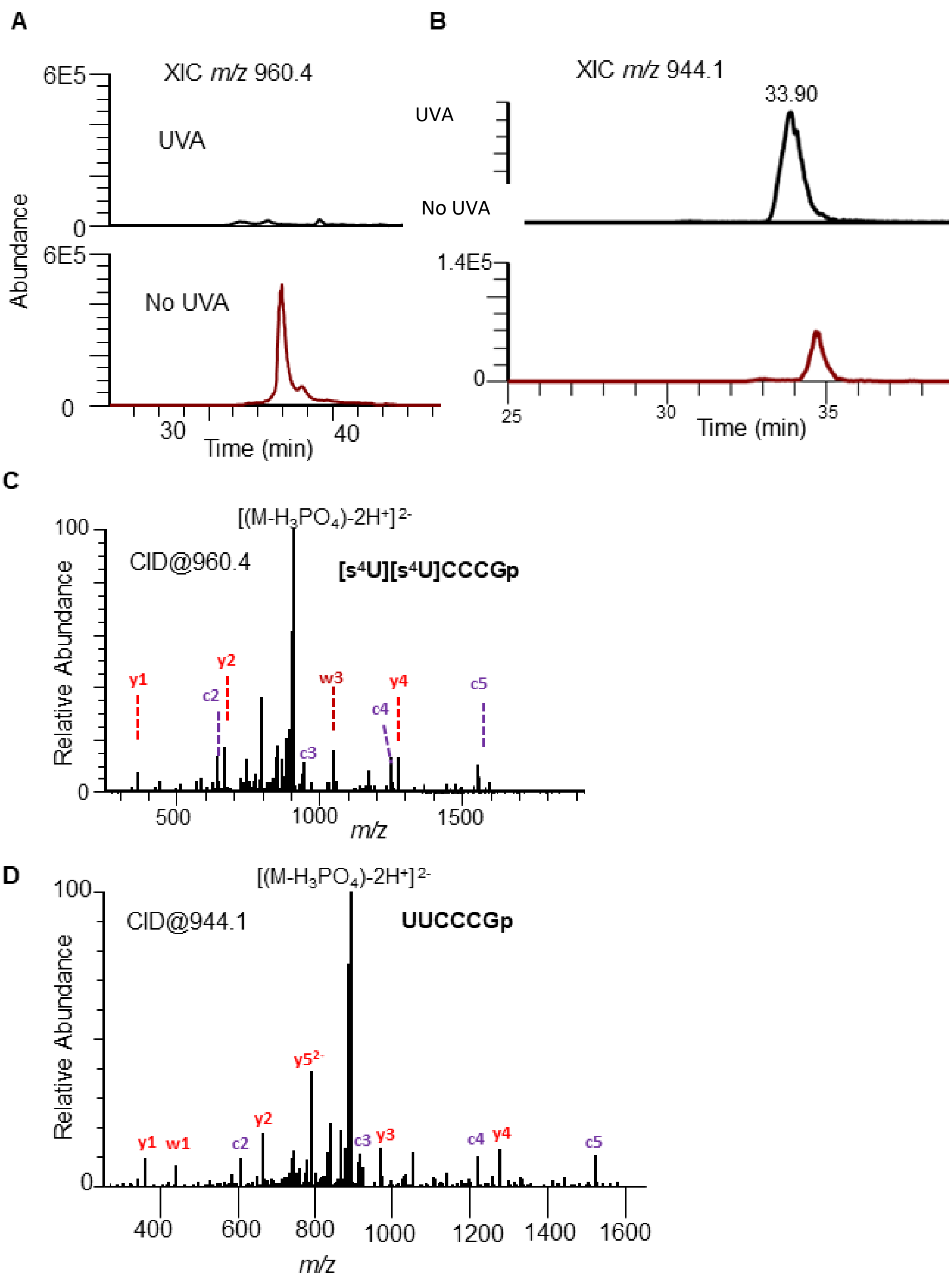

Supplemental Figure S6: Photooxidative degradative loss of **s⁴U** in the UVA exposed tRNA^{Tyr} of *E. coli* **(A)** Extracted ion chromatogram (XIC) for *m/z* 960.4 (z=-2) corresponding to [s^{4}U][s^{4}U]CCCGp (position 8-13) in the UVA exposed and unexposed samples. (**B**) Extracted ion chromatogram (XIC) for *m/z* 944.1 (z=-2) corresponding to UUCCCGp (position 8-13) in the UVA exposed and unexposed samples. (**C**) MS/MS spectrum of *m/z* 960.4 with sequence informative product ion pattern (cn fragment ion series from 5'-end and yn fragment ion series from 3'-end) of the oligomer**. (D)** MS/MS spectrum of *m/z* 944.1 with sequence informative product ion pattern (cn fragment ion series from 5'-end and yn fragment ion series from 3'-end) of the oligomer.

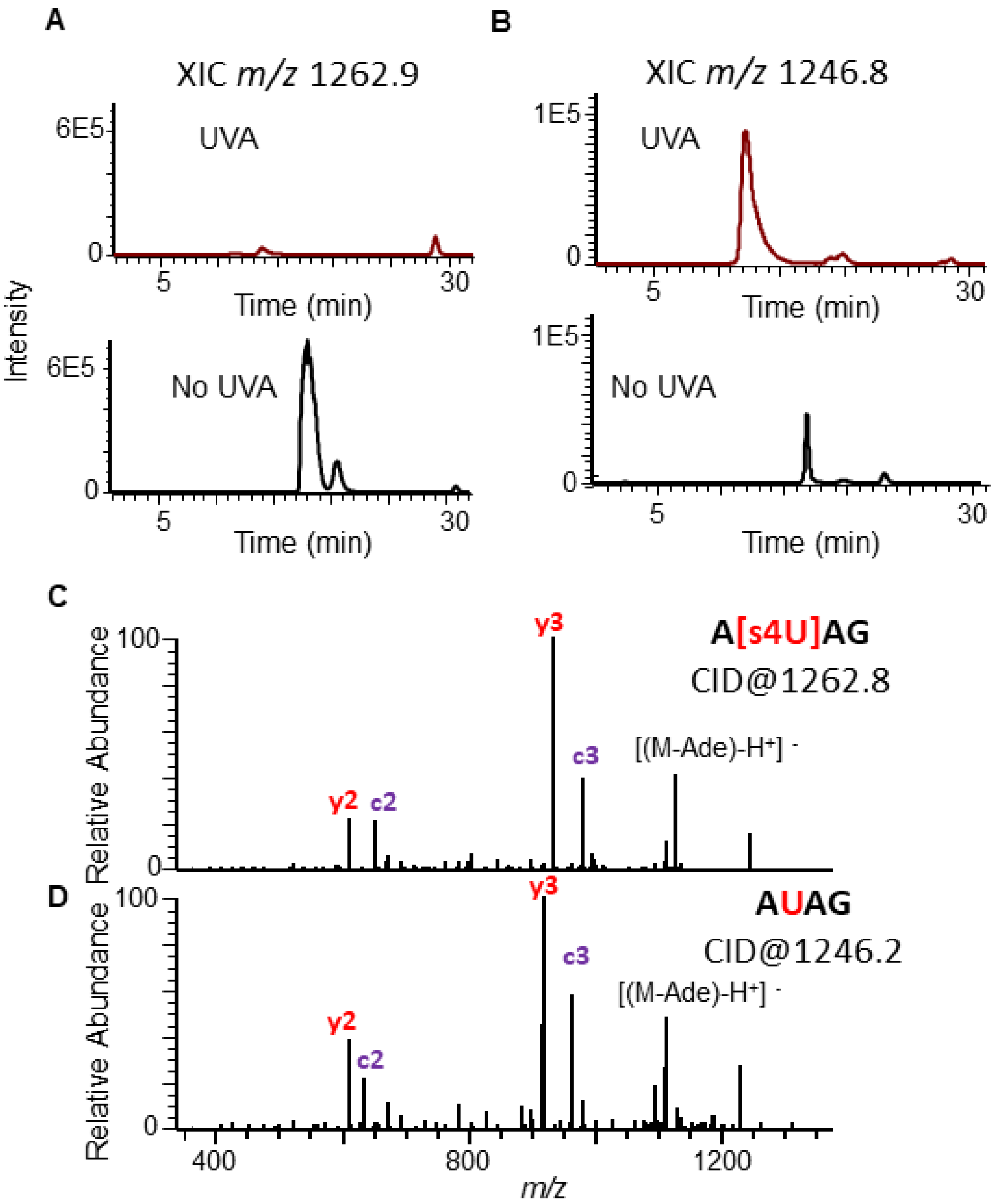

Supplemental Figure S7: Dethiolation of s^4U containing oligonucleotide, A[s^4U]AG following UVA exposure. (A) Extracted ion chromatogram (XIC) for *m/z* 1262.9 corresponding to A[s^4U]AG in the exposed and unexposed samples. (**B**) Extracted ion chromatogram (XIC) for *m/z* 1246.8 corresponding to AUAG in the UVA exposed and unexposed samples. (**C**) MS/MS spectrum of *m/z* 1262.9 with sequence informative product ion pattern (cn fragment ion series from 5'-end and yn fragment ion series from 3'-end) of the oligomer, A[s^4U]AG. (**D**) MS/MS spectrum of *m/z* 1246.2 with sequence informative product ion pattern (cn fragment ion series from 5'-end and yn fragment ion series from 3'-end) of the oligomer, AUAG.

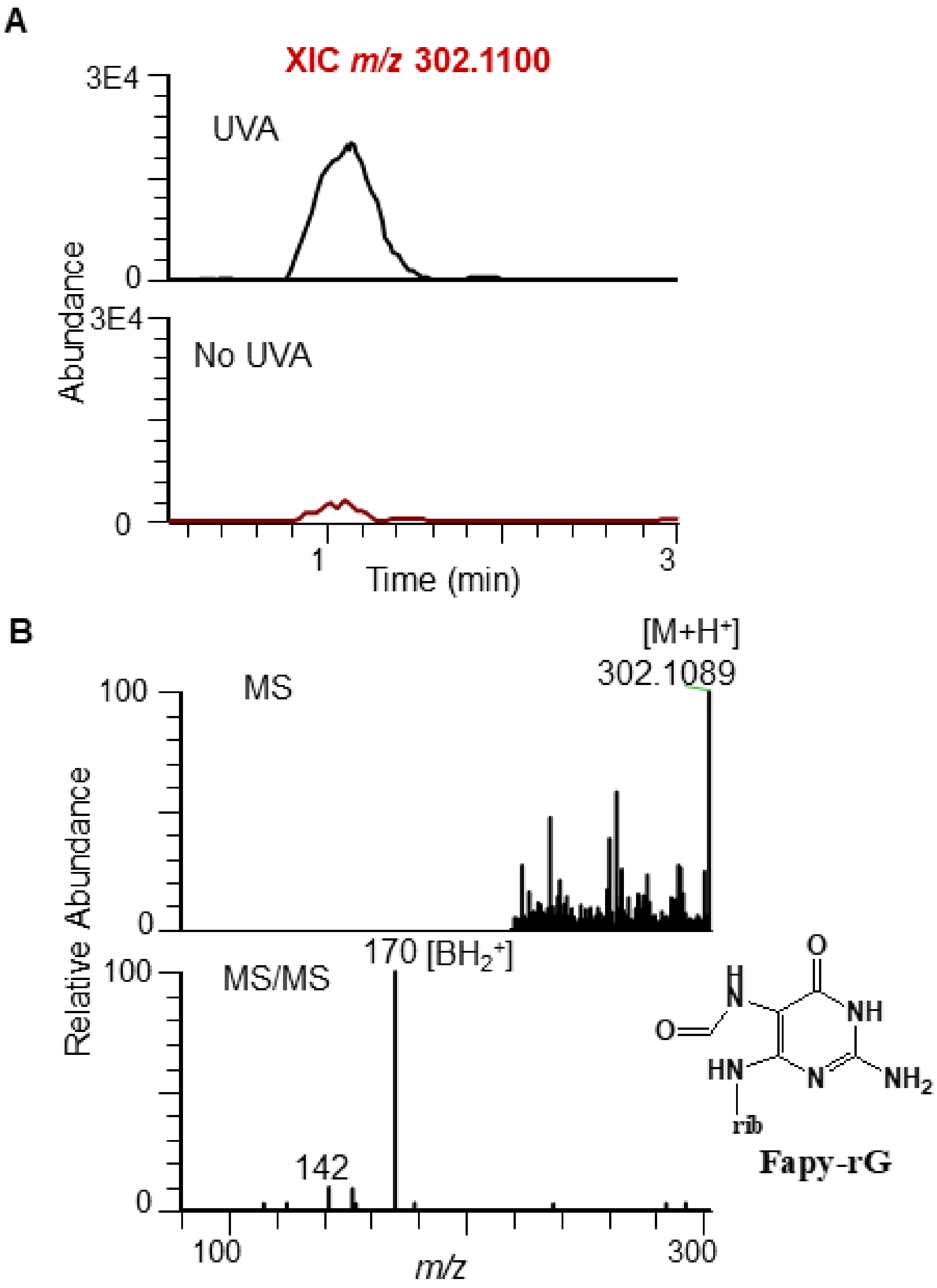

Supplemental Figure S8: Detection of guanosine oxidation product FapyG in the tRNA^{Tyr} following UVA exposure. The nucleoside analysis was performed by high resolution LC-MS using Vanquish UHPLC system coupled with Orbitrap Fusion Lumos (Thermo Scientific) mass spectrometer as described before (Sun, Jora et al. 2018). **A.** Extracted ion chromatogram for *m/z* 302.110 corresponding to FapyG in the UVA exposed sample. **B** Mass spectrum of the XIC at 1.1 min (top panel), and MS/MS spectrum of the molecular ion depicting the signal for Fapy-G nucleobase (bottom panel) are shown.

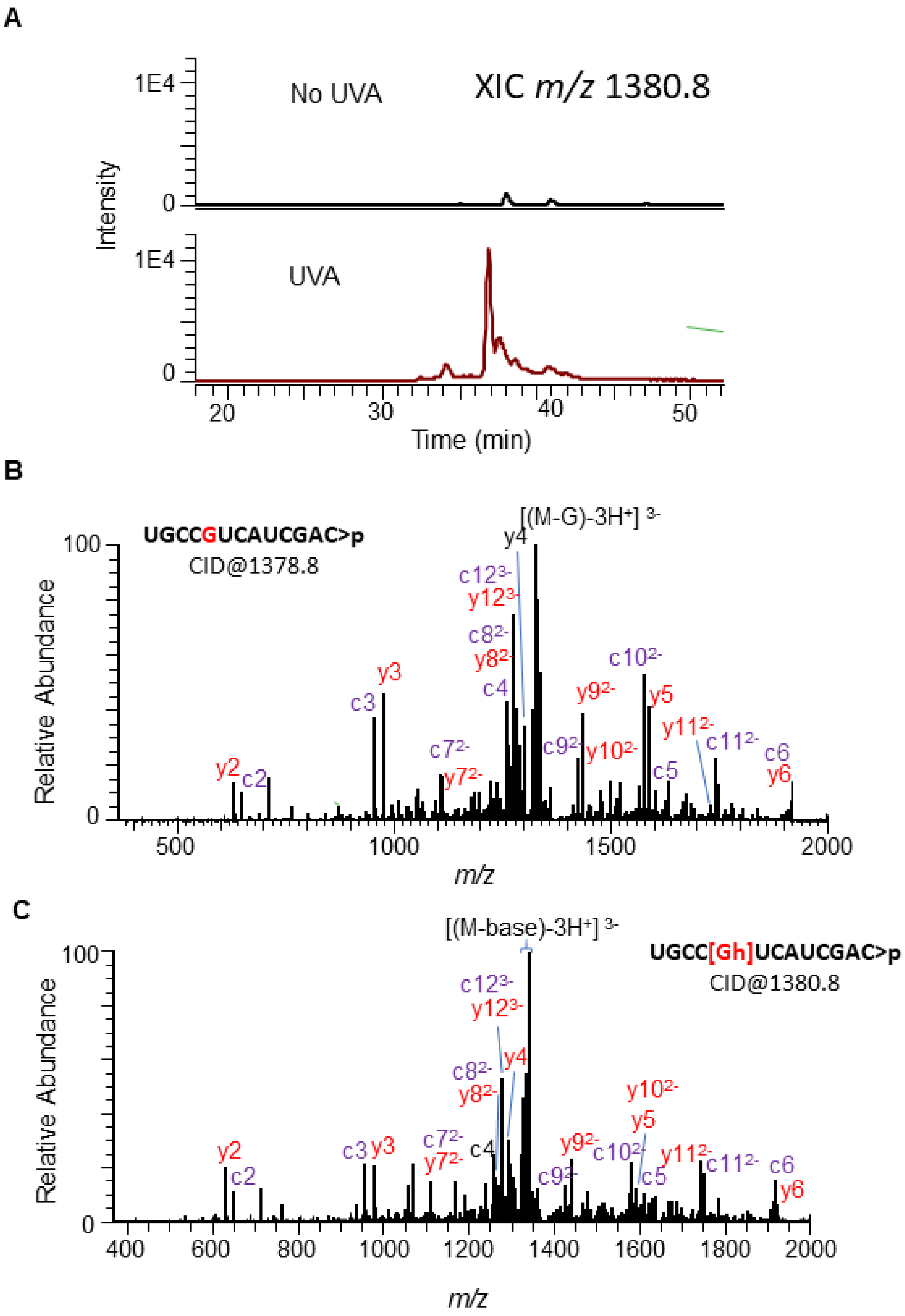

Supplemental Figure S9: Detection of UGCC[Gh]UCAUCGAC>p**,** *m/z* 1380.8 (z=-3, position 41-52) in the UVA exposed tRNA^{Tyr} of *E. coli* by LC-MS/MS. **(A)** Extracted ion chromatogram (XIC) for *m/z* 1380.8 corresponding to UGCC[Gh]UCAUCGAC>p in the UVA exposed and unexposed samples. (**B**) MS/MS spectrum of *m/z* 1378.8 with sequence informative product ion pattern (cn fragment ion series from 5'-end and yn fragment ion series from 3'-end) of the oligomer, UGCCGUCAUCGAC>p(**C**) MS/MS spectrum of *m/z* 1380.8 with sequence informative product ion pattern of the oligomer, UGCC[Gh]UCAUCGAC>p **.**

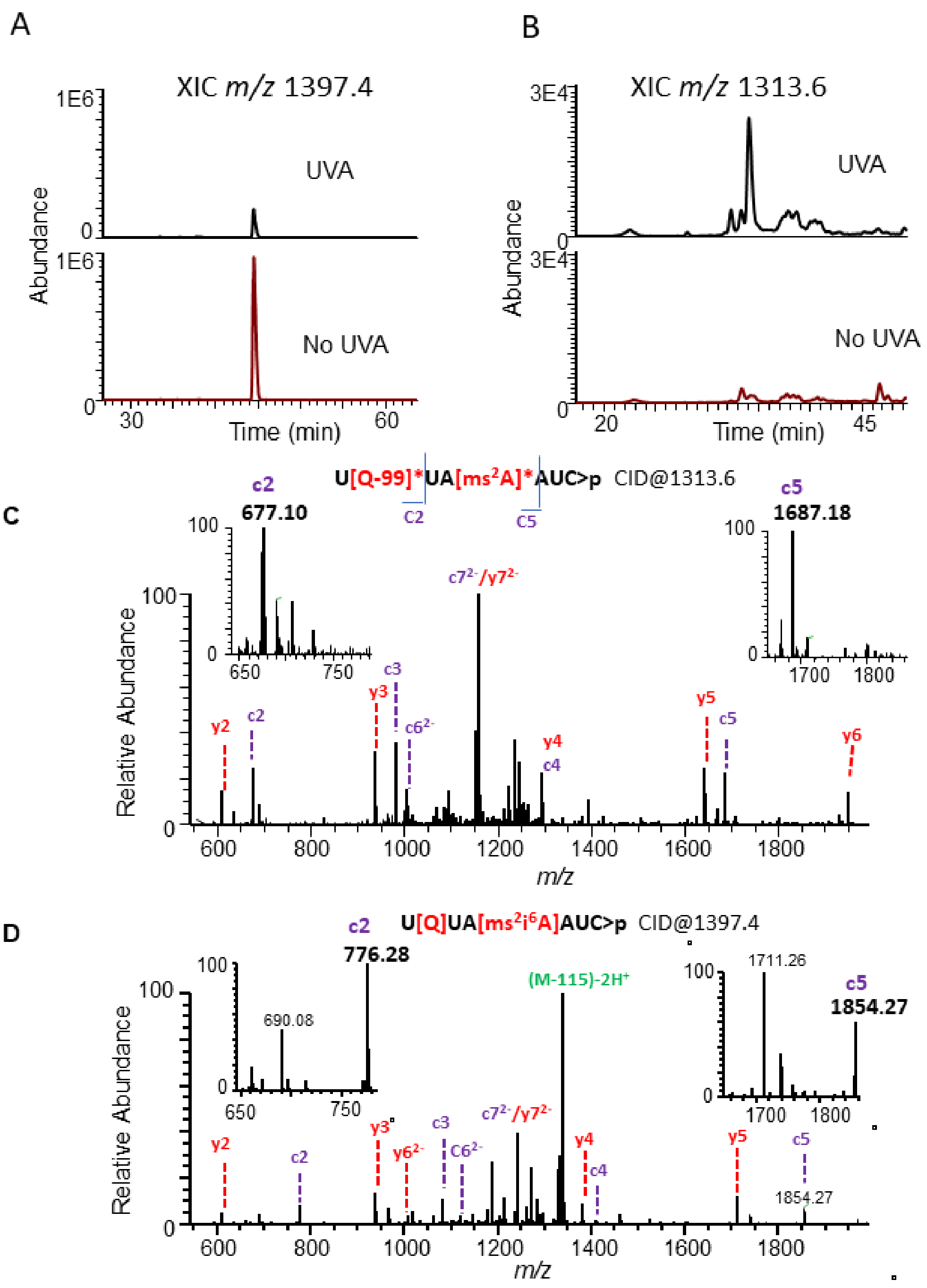

Supplemental Figure S10: Detection of U[Q-99]*ψA[ms^{2}A]*AψC>p *m/z* 1313.6 (z=-2, position 33-40) in the UVA exposed tRNA^{Tyr} of *E. coli* by LC-MS/MS. **(A)** Extracted ion chromatogram (XIC) for *m/z* 1397.4 corresponding to U[Q]ψA[ms^{2}i^{6}A]AψC>p in the UVA exposed and unexposed samples. (**B**) Extracted ion chromatogram (XIC) for *m/z* 1313.6 corresponding to U[Q-99]ψA[ms^{2}A]AψC>p in the UVA exposed and unexposed samples. (C) MS/MS spectrum of *m/z* 1313.6 with sequence informative product ion pattern (cn fragment ion series from 5'-end and yn fragment ion series from 3'-end) of the oligomer, U[Q-99]ψA[ms^{2}A]AψC>p (D) MS/MS spectrum of *m/z* 1397.4 with sequence informative product ion pattern of the oligomer, U[Q]ψA[ms^{2}i^{6}A]AψC>p **.** Note the difference in mass shift of c2 and c5 fragment ions in UVA and no UVA exposed samples.

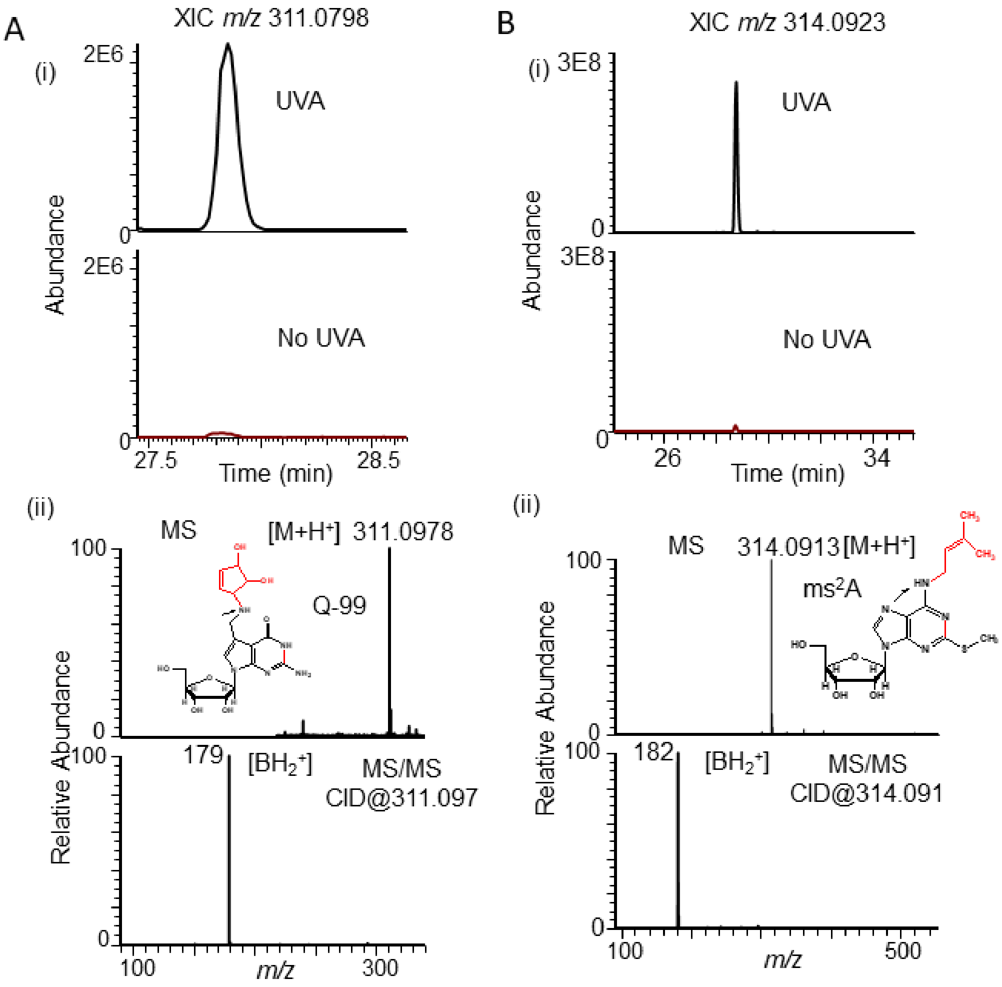

Supplemental Figure S11: Detection of photooxidative degradation products of queuosine and ms^{2}i^{6}A. **(A)** Detection of the photooxidative degradation product of queuosine in the tRNA^{Tyr} following UVA exposure. The nucleoside analysis was performed by high resolution LC-MS using a Vanquish UHPLC system coupled to an Orbitrap Fusion Lumos (Thermo Scientific) mass spectrometer as described before (Sun, Jora et al. 2018). (i) Extracted ion chromatogram for *m/z* 311.079 corresponding to Q-99 in the UVA exposed sample. The cleavage point that leads to loss of 99 Da from Q (410.1597) is shown. (ii) Mass spectrum of the XIC at 27.9 min (top panel), MS/MS spectrum of the molecular ion depicting the signal for Q-99 nucleobase (bottom panel). (**B**) Detection of the degradation product of ms^{2}A in the tRNA^{Tyr} following UVA exposure. The cleavage point that leads to loss of i^6 group (loss of 68.054 Da) from ms^{2}i^{6}A is shown. (i) Extracted ion chromatogram for *m/z* 314.092 corresponding ms^{2}A in the UVA exposed sample. (ii) Mass spectrum of the XIC at 27.2 min depicting the altered modification, the site of cleavage on ms^{2}i^{6}A (381.1471 Da) leading to ms^{2}A formation is shown (top panel) and MS/MS spectrum of the molecular ion *m/z* 314.092 leading to the formation of ms^{2}A nucleobase due to loss of ribose sugar is shown (bottom panel).

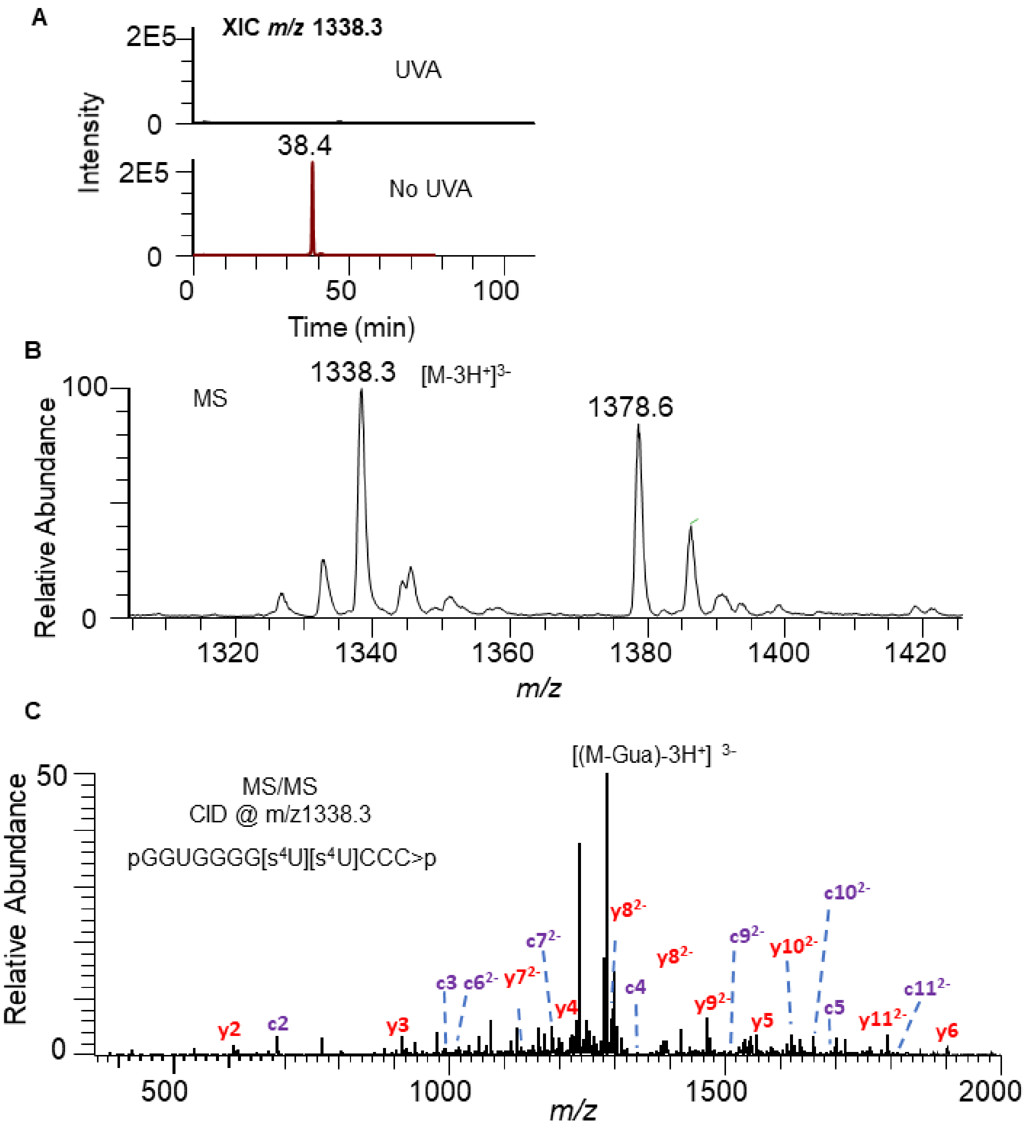

Supplemental Figure S12: Dethiolation of [s^{4}U] in tRNA^{Tyr} isolated from UVA-exposed *E. coli* cells. (**A**) Extracted ion chromatogram (XIC) for *m/z* 1338.3 corresponding to pGGUGGGG[s^{4}U][s^{4}U]CCC>p in the UVA exposed and unexposed samples. (**B**) Mass spectrum of the XIC at 38.4 min depicting the molecular ion. (C) MS/MS spectrum of *m/z* 1338.3 with sequence informative product ion pattern (cn fragment ion series from 5'-end and yn fragment ion series from 3'-end) of the oligomer, pGGUGGGG[s^{4}U][s^{4}U]CCC>p **.**

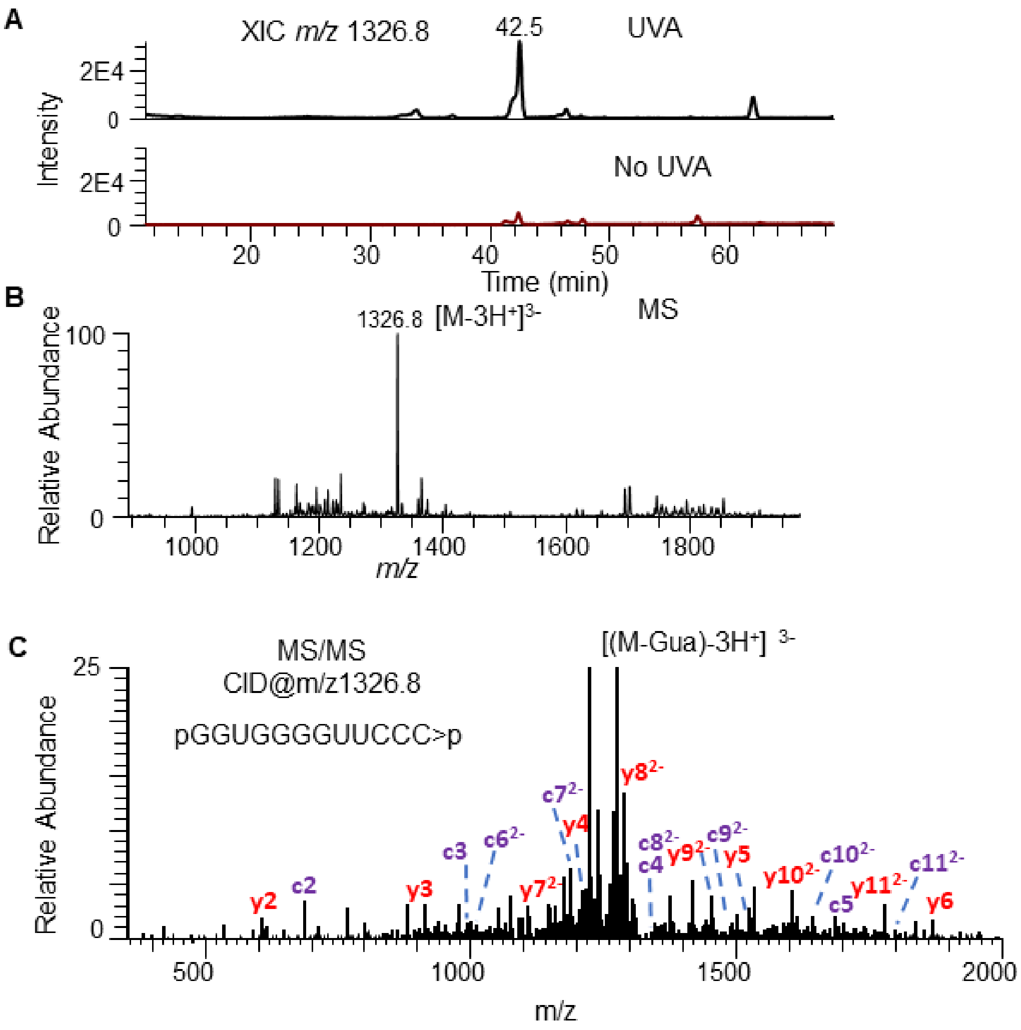

Supplemental Figure S13: Detection of dethiolated oligomer pGGUGGGGUUCCC>p in tRNA^{Tyr} isolated from UVA-exposed *E. coli* cells. (**A)** Extracted ion chromatogram (XIC) for *m/z* 1326.8 corresponding to pGGUGGGGUUCCC>p in the UVA exposed and unexposed samples. (**B**) Mass spectrum of the XIC at 42.5 min depicting the molecular ion. (C) MS/MS spectrum of *m/z* 1326.8 with sequence informative product ion pattern (cn fragment ion series from 5'-end and yn fragment ion series from 3'-end) of the oligomer, pGGUGGGGUUCCC>p **.**

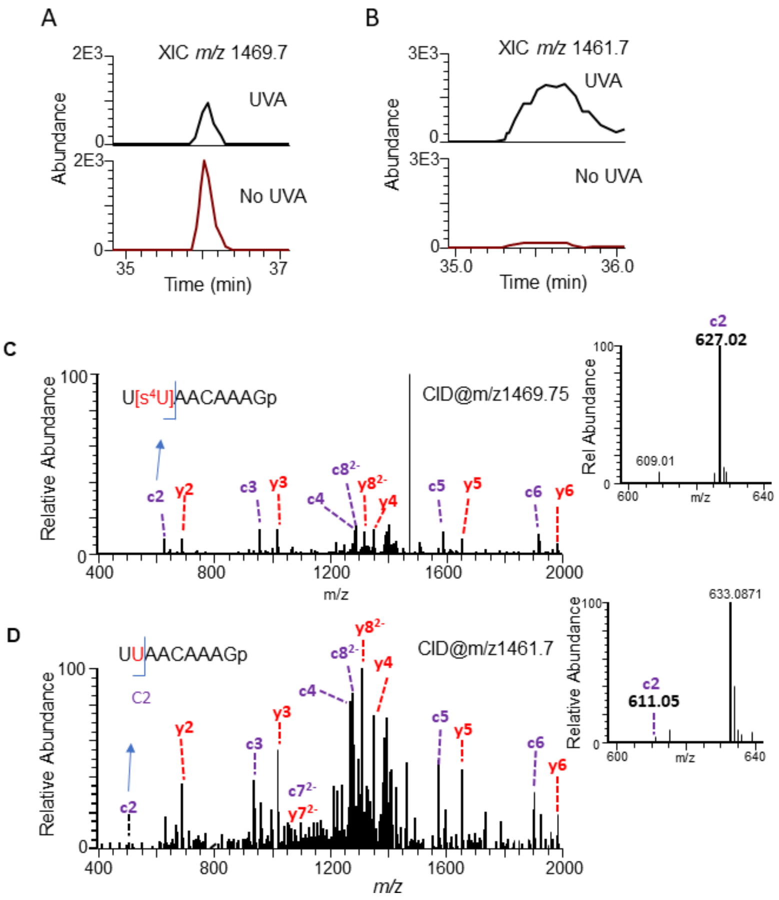

Supplemental Figure S14: Decreased levels of [s^{4}U]-containing oligonucleotide U[s^{4}U]AACAAAGp from **tRNA^{Cys}** in the UVA exposed *E. coli* cells by LC-MS/MS. **(A)** Extracted ion chromatogram (XIC) for *m/z* 1469.7 corresponding to **U**[s^{4}U]AACAAAGp (position 7-15**)** in the UVA exposed and unexposed samples. (**B**) Extracted ion chromatogram (XIC) for *m/z* 1461.7 corresponding to UUAACAAAGp in the UVA exposed and unexposed samples. (C) MS/MS spectrum of *m/z* 1469.7 with sequence informative product ion pattern (cn fragment ion series from 5'-end and yn fragment ion series from 3'-end) of the oligomer - U[s^{4}U]AACAAAGp (D) MS/MS spectrum of *m/z* 1469.7 with sequence informative product ion pattern of the oligomer - UUAACAAAGp**.** Note the difference in mass shift of c2 fragment ions in UVA and no UVA exposed samples.

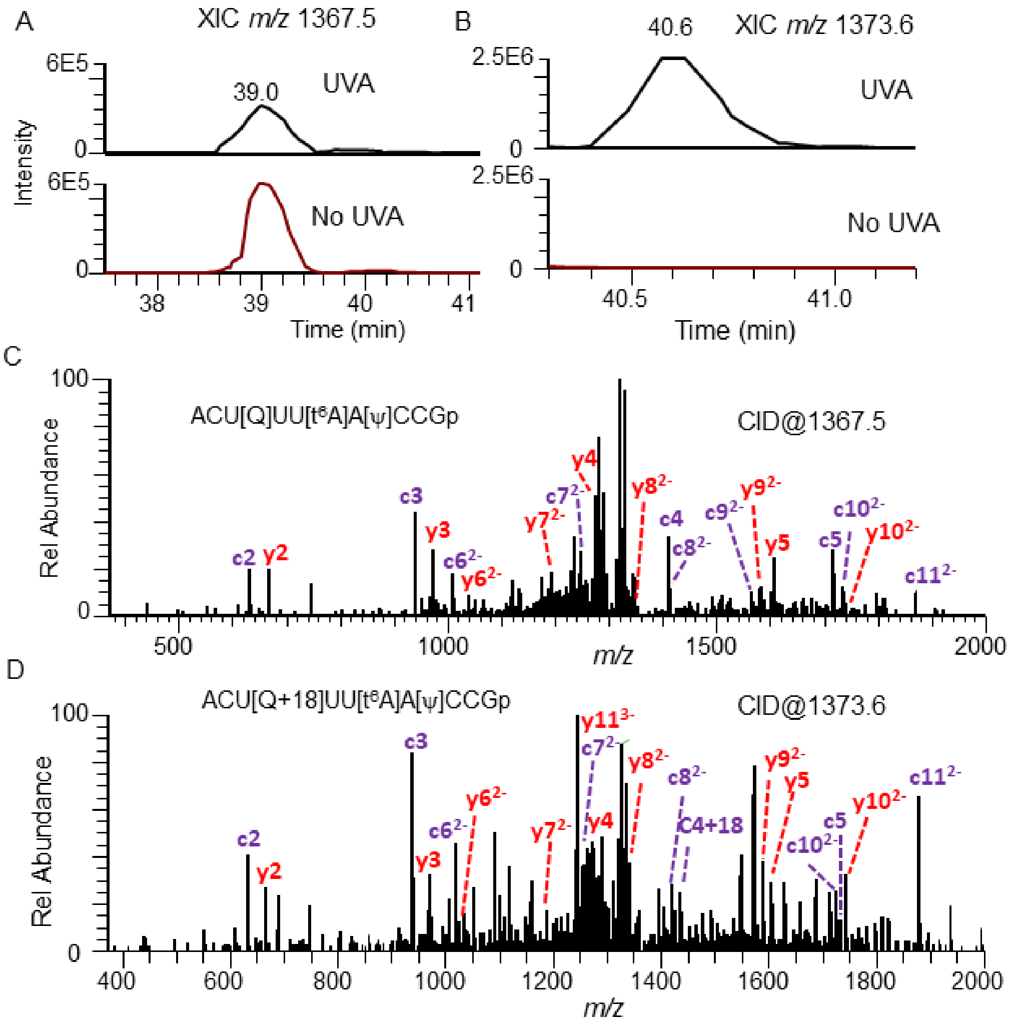

Supplemental Figure S15: Decrease in levels of Q-containing oligonucleotide ACU[Q]UU[t^{6}A]AψCCGp from tRNA^{Asn} in the UVA exposed and unexposed *E. coli* cells. **(A)** Extracted ion chromatogram (XIC) for *m/z* 1367.5 corresponding to ACU[Q]UU[t^{6}A]AψCCGp (position 7-15**)** in the UVA exposed and unexposed samples. (**B**) Extracted ion chromatogram (XIC) for *m/z* 1373.6 corresponding to ACU[Q+18]UU[t^{6}A]AψCCGp in the UVA exposed and unexposed samples. (C) MS/MS spectrum of *m/z* 1367.5 with sequence informative product ion pattern (cn fragment ion series from 5'-end and yn fragment ion series from 3'-end) of the oligomer ACU[Q]UU[t^{6}A]AψCCGp (D) MS/MS spectrum of *m/z* 1373.6 with sequence informative product ion pattern of the oligomer ACU[Q+18]UU[t^{6}A]AψCCGp.

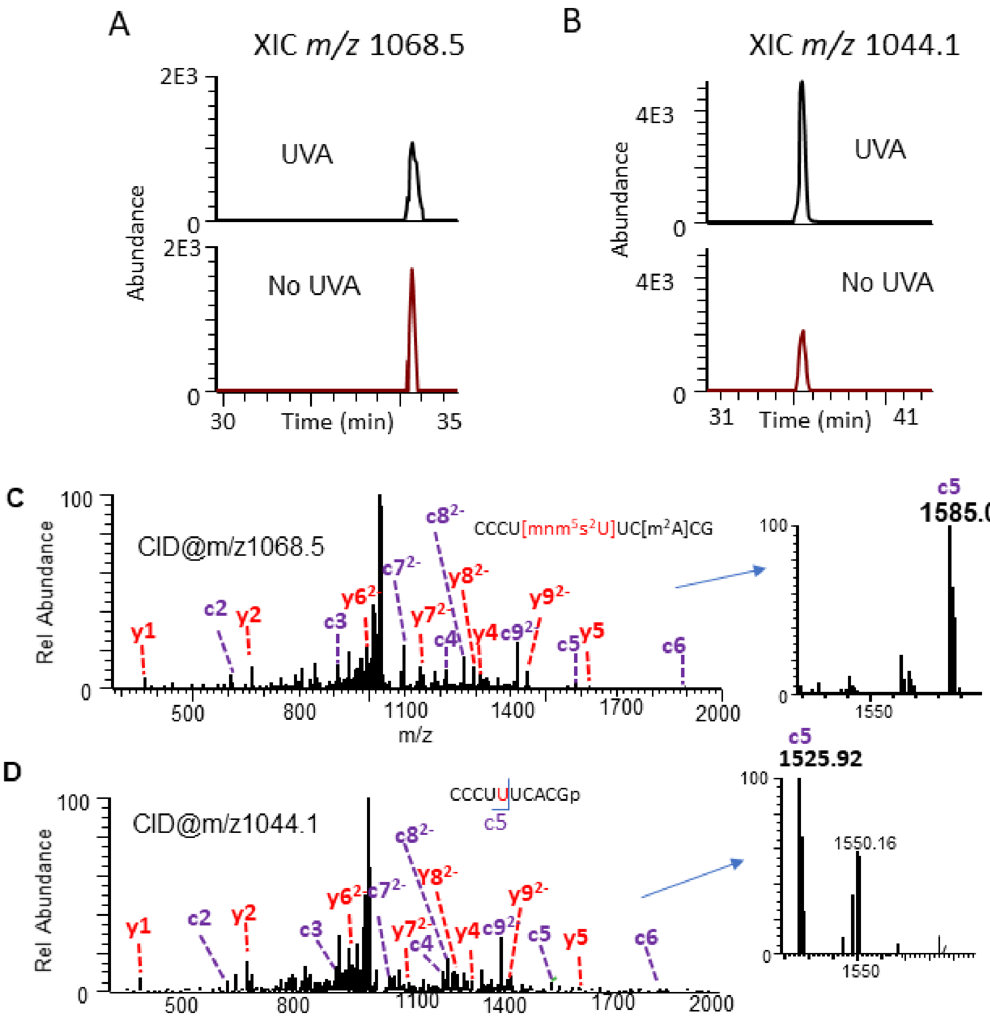

Supplemental Figure S16 Decreased levels of mnm^{5}s^{2}U containing oligonucleotide of tRNA^{Glu} in the UVA exposed *E. coli* cells by LC-MS/MS. **(A)** Extracted ion chromatogram (XIC) for *m/z* 1068.5 corresponding to CCCU[mnm^{5}s^{2}U]UC[m^{2}A]CGp (position 30-39**)** in the UVA exposed and unexposed samples. (**B**) Extracted ion chromatogram (XIC) for *m/z* 1044.1 corresponding to CCCUUUCACGp in the UVA exposed and unexposed samples. (C) MS/MS spectrum of *m/z* 1068.5 with sequence informative product ion pattern (cn fragment ion series from 5'-end and yn fragment ion series from 3'-end) of the oligomer - , CCCU[mnm^{5}s^{2}U]UC[m^{2}A]CGp (D) MS/MS spectrum of *m/z* 1068.5 with sequence informative product ion pattern of the oligomer - CCCUUUCACGp**.** Note the difference in mass shift of c5 fragment ions in UVA and no UVA exposed samples

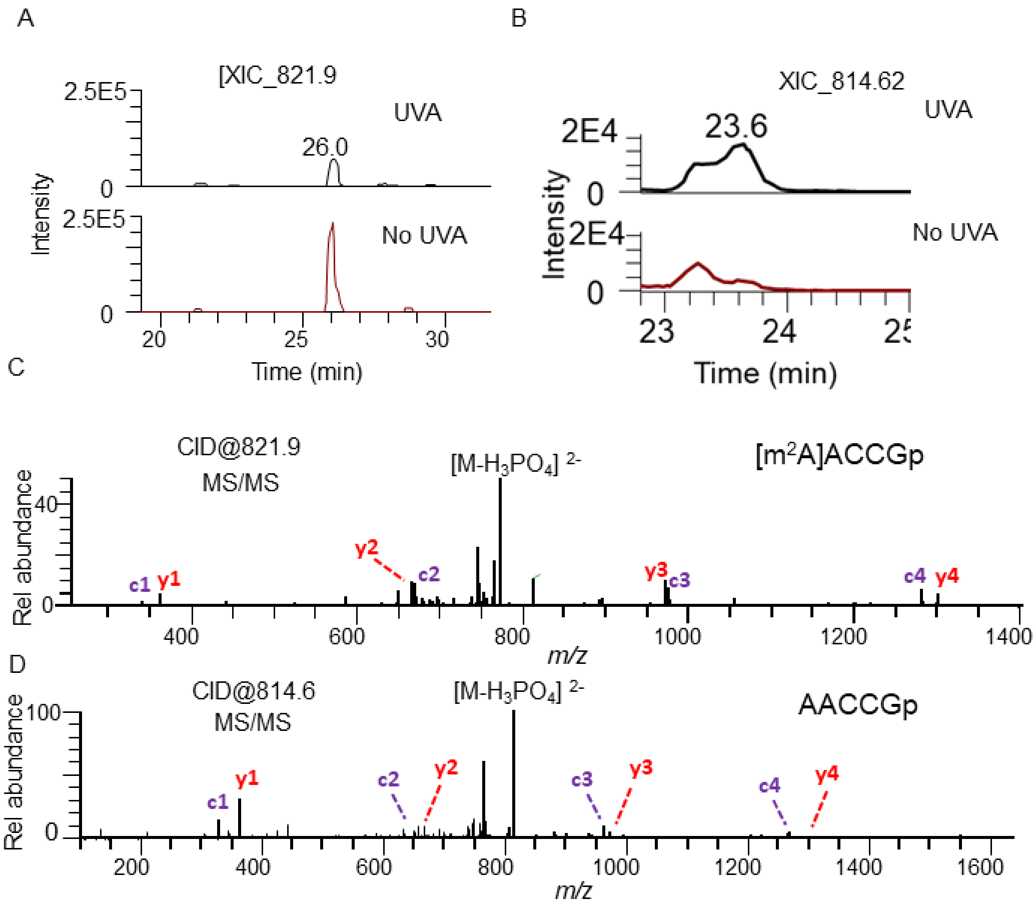

Supplemental Figure S17: Decreased levels of m^{2}A containing oligonucleotide from tRNA$^{Arg(ICG)}$ in the UVA exposed compared to unexposed *E. coli* cells **(A)** Extracted ion chromatogram (XIC) for *m/z* 821.9 corresponding to [m^{2}A]ACCGp (position 37-41**)** in the UVA exposed and unexposed samples. (**B**) Extracted ion chromatogram (XIC) for *m/z* 814.6 corresponding to AACCGp (position 37-41**)** in the UVA exposed and unexposed samples. Mass spectrum of XIC at 29.8 min (**C**) MS/MS spectrum of *m/z* 821.9 with sequence informative product ion pattern of the oligomer, [m^{2}A]ACCGp**. (D)** MS/MS spectrum of *m/z* 814.6 with sequence informative product ion pattern of the oligomer AACCGp.

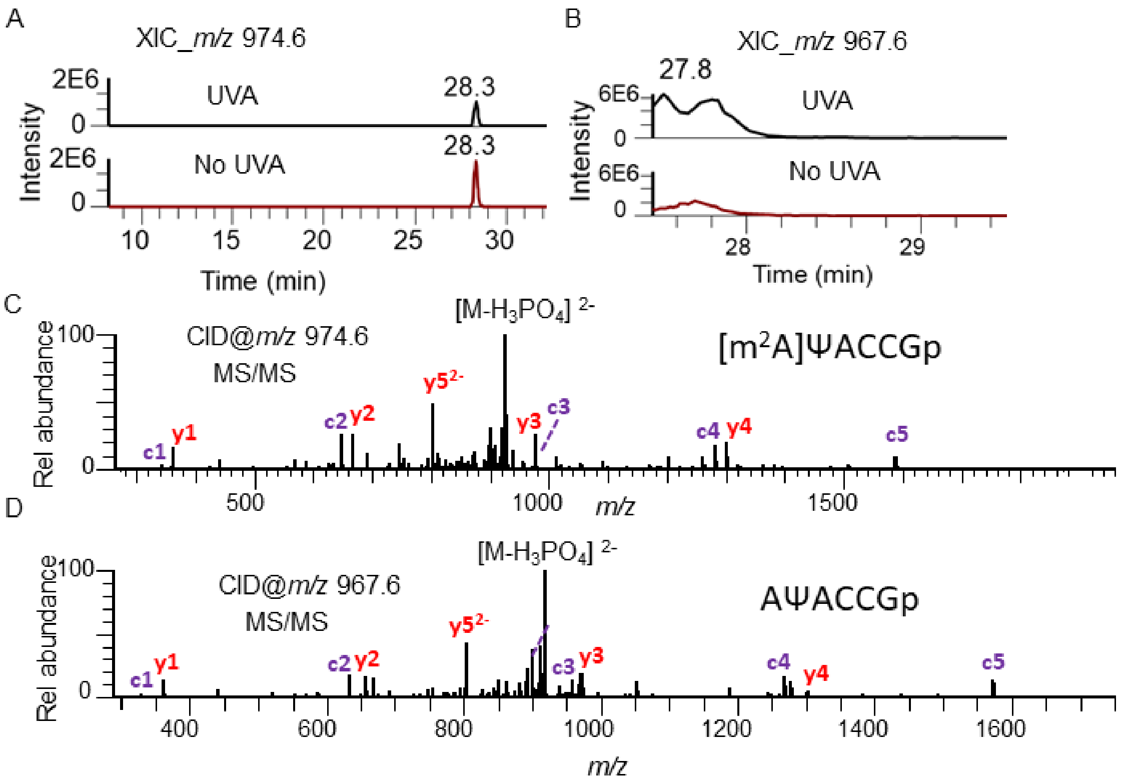

Supplemental Figure S18: Decreased levels of m^{2}A containing oligonucleotide from tRNA$^{Gln(UUG)}$ in the UVA exposed compared to unexposed *E. coli* cells **(A)** Extracted ion chromatogram (XIC) for *m/z* 974.63 corresponding to [m^{2}A]ψACCGp (position 37-41**)** in the UVA exposed and unexposed samples. (**B**) Extracted ion chromatogram (XIC) for *m/z* 967.62.6 corresponding to AψACCGp (position 37-41**)** in the UVA exposed and unexposed samples. (**C**) MS/MS spectrum of *m/z* 974.63 with sequence informative product ion pattern of the oligomer, [m^{2}A]ψACCGp**. (D)** MS/MS spectrum of *m/z* 967.626 with sequence informative product ion pattern of the oligomer AψACCGp.

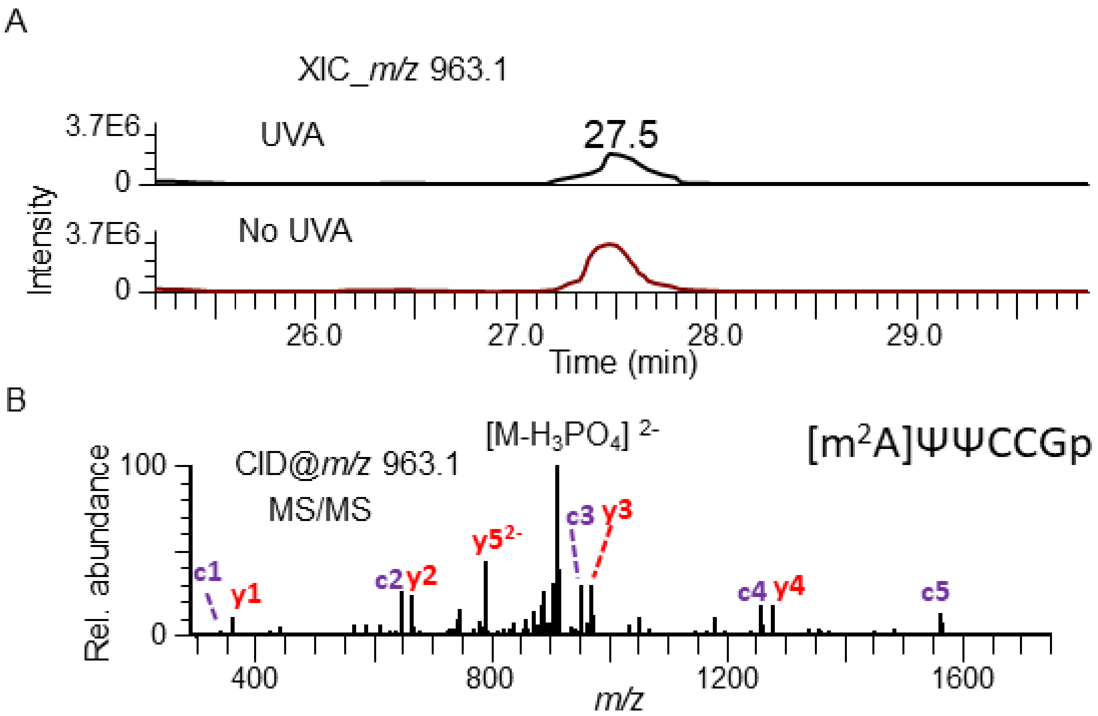

Supplemental Figure S19: Decreased levels of m^{2}A containing oligonucleotide of tRNA$^{Gln(CUG)}$ in the UVA exposed and unexposed *E. coli* cells **(A)** Extracted ion chromatogram (XIC) for *m/z* 963.1 corresponding to **[**m^{2}A]ψψCCGp (position 37-42**)** in the UVA exposed and unexposed samples. **(B)** MS/MS spectrum of *m/z* 963.1 with sequence informative product ion pattern of the oligomer, [m^{2}A]ψψCCGp**.** Detection of its unmodified form is confounded by the presence of isomeric sequences.

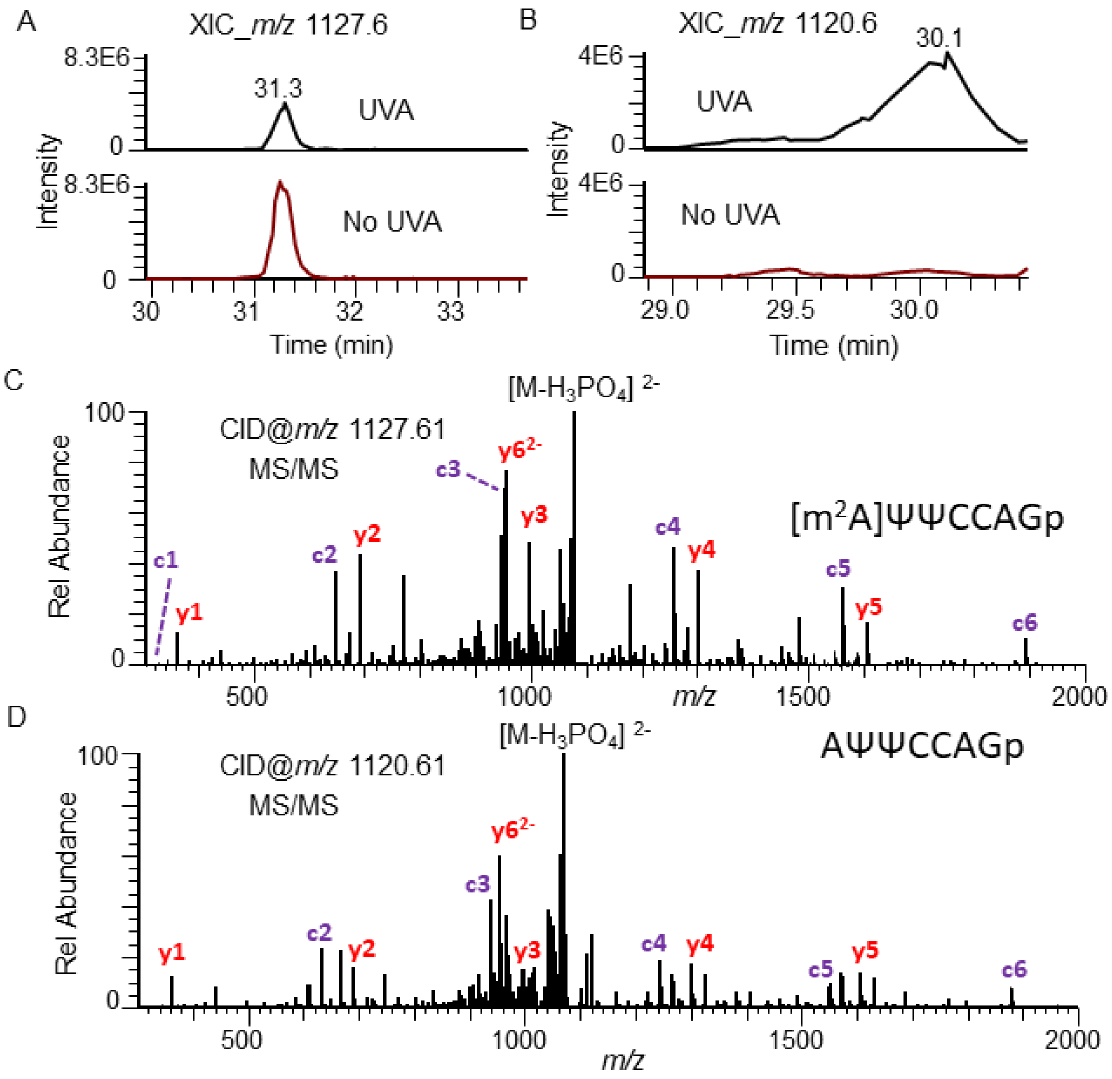

Supplemental Figure S20: Decreased levels of m^{2}A containing oligonucleotide from tRNA$^{His(GUG)}$ in the UVA exposed and unexposed *E. coli* cells **(A)** Extracted ion chromatogram (XIC) for *m/z* 1127.61 corresponding to [m^{2}A]ψψCCAGp (position 37-43**)** in the UVA exposed and unexposed samples. (**B**) Extracted ion chromatogram (XIC) for *m/z* 1120.6 corresponding to AψψCCAGp (position 37-43**)** in the UVA exposed and unexposed samples. (**C**) MS/MS spectrum of *m/z* 1127.61 with sequence informative product ion pattern of the oligomer, [m^{2}A]ψψCCAGp**. (D)** MS/MS spectrum of *m/z* 1120.61 with sequence informative product ion pattern of the oligomer AψψCCAGp.

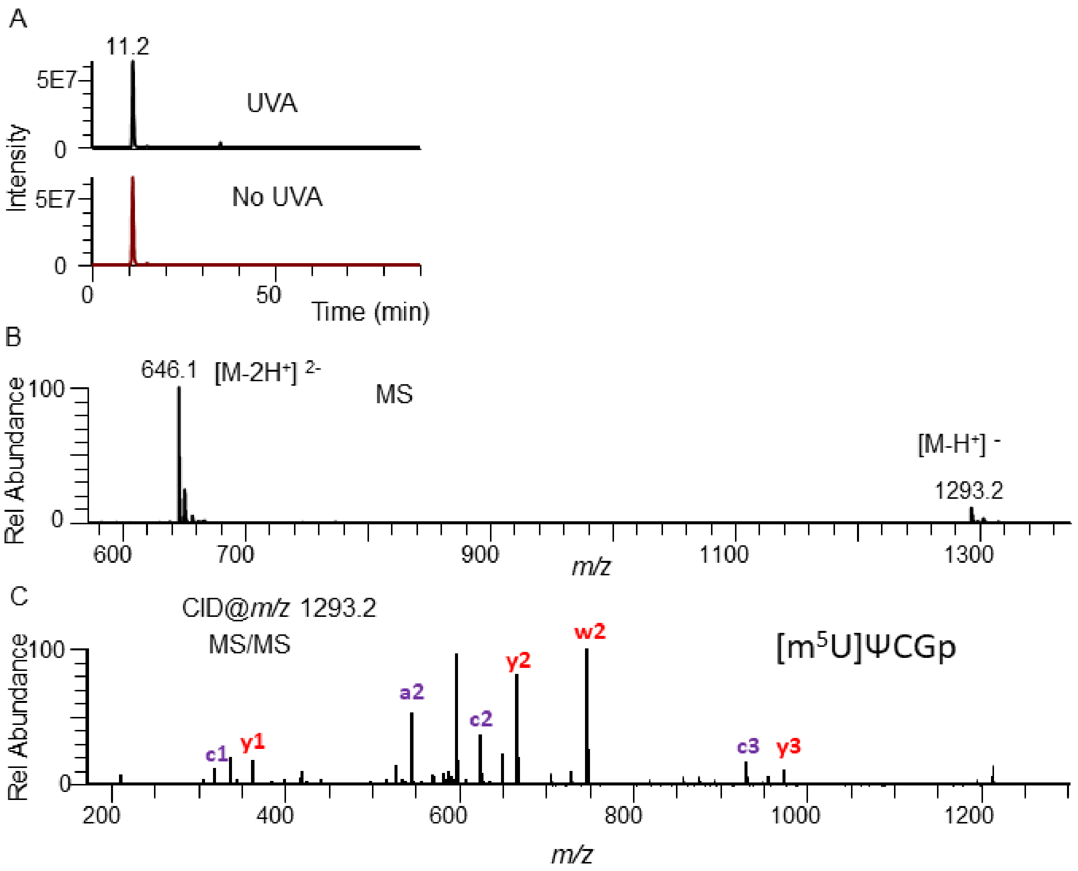

Supplemental Figure S21: Levels of [m^{5}U]ΨCGp are unaffected between the UVA exposed and unexposed *E. coli* cells **(A)** Extracted ion chromatogram (XIC) for *m/z* 1293.2 corresponding to [m^{2}A]ψCGp arising from the TψC-loop of several tRNAs in the UVA exposed and unexposed samples. (**B**) Mass spectrum of the XIC at 11.2 min. (**C**) MS/MS spectrum of *m/z* 1293.1 with sequence informative product ion pattern of the oligomer, [m^{2}A]ψCGp**.**
